# Rapid maxillary expansion outcomes according to midpalatal suture maturation levels

**DOI:** 10.1186/s40510-019-0278-9

**Published:** 2019-07-15

**Authors:** Gülşilay Sayar, Delal Dara Kılınç

**Affiliations:** 1 Department of Orthodontics, School of Dentistry, Istanbul Medipol University, Atatürk Bulvarı No: 27, 34083 Unkapanı–Fatih/Istanbul, Turkey; 2 Department of Orthodontics, School of Dental Medicine, Bahçeşehir University, Istanbul, Turkey

**Keywords:** Rapid maxillary expansion, Cone beam computed tomography, Midpalatal suture, Orthodontics

## Abstract

**Background:**

This study aims to compare the relationship between skeletal and dental outcomes of rapid maxillary expansion (RME) treatment on cone beam computed tomography (CBCT) images between pre-pubertal peak (pre-peak) and post-pubertal peak (post-peak) patients. The null hypothesis was that there was no difference in the outcomes of RME treatment between the groups.

**Methods:**

Thirty-two patients who underwent RME treatment were classified according to midpalatal suture maturation levels and then divided into two groups as pre-peak and post-peak. Skeletal and dental measurements were performed on the CBCT images at T0 (pre-treatment stage) and at T1 (post-treatment stage). Paired sample *t* test was used to evaluate normally distributed data and *P* < 0.05 was taken as the significance level.

**Results:**

There were significant differences between T0 and T1 within the groups, but the changes between the pre-peak and post-peak patient groups were not statistically significant.

**Conclusion:**

Non-significant changes were found between the two groups, and the null hypothesis was excepted.

## Introduction

Maxillary transversal deficiency can be associated with every types of sagittal malocclusion [1, 2]. Orthodontists prefer to widen the maxillary basal bone by using various types of maxillary expansion devices such as bonded, banded, or hybrid expanders [3–5].

Skeletal and dental effects were widely investigated in the literature [1, 3–6], but there is still limited information present about the prediction of maxillary expansion results [7]. Clinical outcomes can be different from the pre-treatment prediction. The biological response of midpalatal suture, skeletal tissues, and dental tissues to rapid maxillary expansion was traditionally reported as it was related to patient’s chronological age. As a result, age is usually accepted as the determining factor while planning maxillary expansion.

In cadaveric studies, it was shown that midpalatal suture maturation could be observed as non-mature even in adults; therefore, age was not accepted as the determinant factor in maturation of the midpalatal suture [8–10]. Besides the treatment method, at the beginning of the treatment, it is very important for an orthodontist to be able to predict whether the enlargement will be more dental than skeletal, or contrary. Highly matured midpalatal suture can be seen even though in young patients, and some kind of complications like pain, alveolar bending, and recession in gingiva or failures such as absence of sutural opening debonding of the expander may be observed during rapid maxillary expansion (RME) treatment [11–13]. Orthopedic correction of maloclussions, as well as RME treatment, is more easily and effectively performed in puberty. With an exception of individual differences, it is known that increase of interdigitation in the midpalatal suture causes a decrease in response to skeletal expansion especially after puberty [8, 14, 15].

Using hand-wrist radiographs [16], cervical vertebral maturation (CVM) method [17] and statural height increase are reliable methods of evaluating skeletal maturation [18]. A new approach to determine skeletal palatal sutural maturation was introduced by Angelieri et al. [19] which was based on evaluation of the phases of midpalatal suture maturation on cone beam computed tomography (CBCT) images.

In the light of the fact that skeletal maturation level affects the biological response to rapid maxillary expansion, the aim of this study was to investigate the relationship between skeletal and dental outcomes of RME treatment between pre-peak and post-peak patients who were classified according to midpalatal suture maturation levels.

## Materials and methods

Thirty-two patients (11 males, 21 females) between 10 and 18 years of age were included in this retrospective study. Twenty-eight patients (14 patients in each group) were found as sufficient to have the power 80% with a 95% confidence interval (CI) and an α of 0.05 to find a meaningful difference of 1 mm in interdental width. The Human Ethics Committee of Istanbul Medipol University approved this study with the approval number 10840098-604.01.01-E.53516 (19.12.2018-712). Skeletal maturation of each patient was assessed by using the midpalatal suture maturation (MSM) method [19] which was performed on CBCT images. Following this, the same subjects were divided into two groups as pre-peak (stage A + B + C) and post-peak (stage D + E) according to the midpalatal maturation stages. The pre-peak group consisted 18 patients (7 males, 11 females), and post-peak group consisted 14 patients (4 males, 10 females).

The CBCT images of the patients were taken at T0 (just before the application of RME device) and T1 (post-treatment stage, at the 6-month retention period, just after the debonding of the device). Inclusion criteria of this study were as follows: patients who have maxillary bilateral transverse deficiency, no craniofacial disorder, and no previous orthodontic treatment were included in the study.

CBCT images were taken by using the i-CAT® dental CBCT device (model 17-19, Imaging Sciences International, Hatfield, PA, USA). The exposure settings were as follows: 120 kV, 5.0 mA for 26.9 s, and voxel size of 0.2 mm. Field of view of the images was 23 × 17 cm. The dataset was evaluated by using the software of iCAT® device.

### Treatment protocol

The routine treatment protocol of RME in this study was as follows: A bonded type of RME device with a hyrax expansion screw (hyrax®, Dentaurum, Ispringen, Germany) was bonded to the teeth with a glass ionomer luting cement (KetacTM Cem radiopaque, 3 M ESPE, Neuss, Germany). The first activation of the bonded RME was performed in the clinic, and then the patients were told to turn the screw 2 turns per day. One week after the first appointment, a control was carried. After 2 weeks of activation (14 days), a 2-mm overcorrection which lasted 5 days was performed, so 3 weeks of activation in total was obtained. Activation numbers of screw were the same for all of the patients. Retention phase was 6 months, and during this period, the RME appliance was left in the mouth.

### CBCT evaluation and method error

**Fig. 1:**
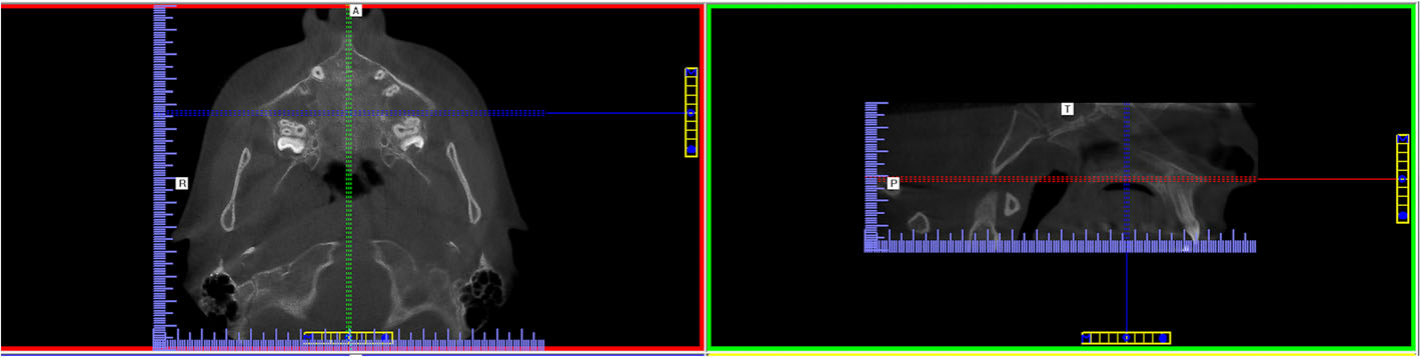
Orientation of the software

**Fig. 2:**
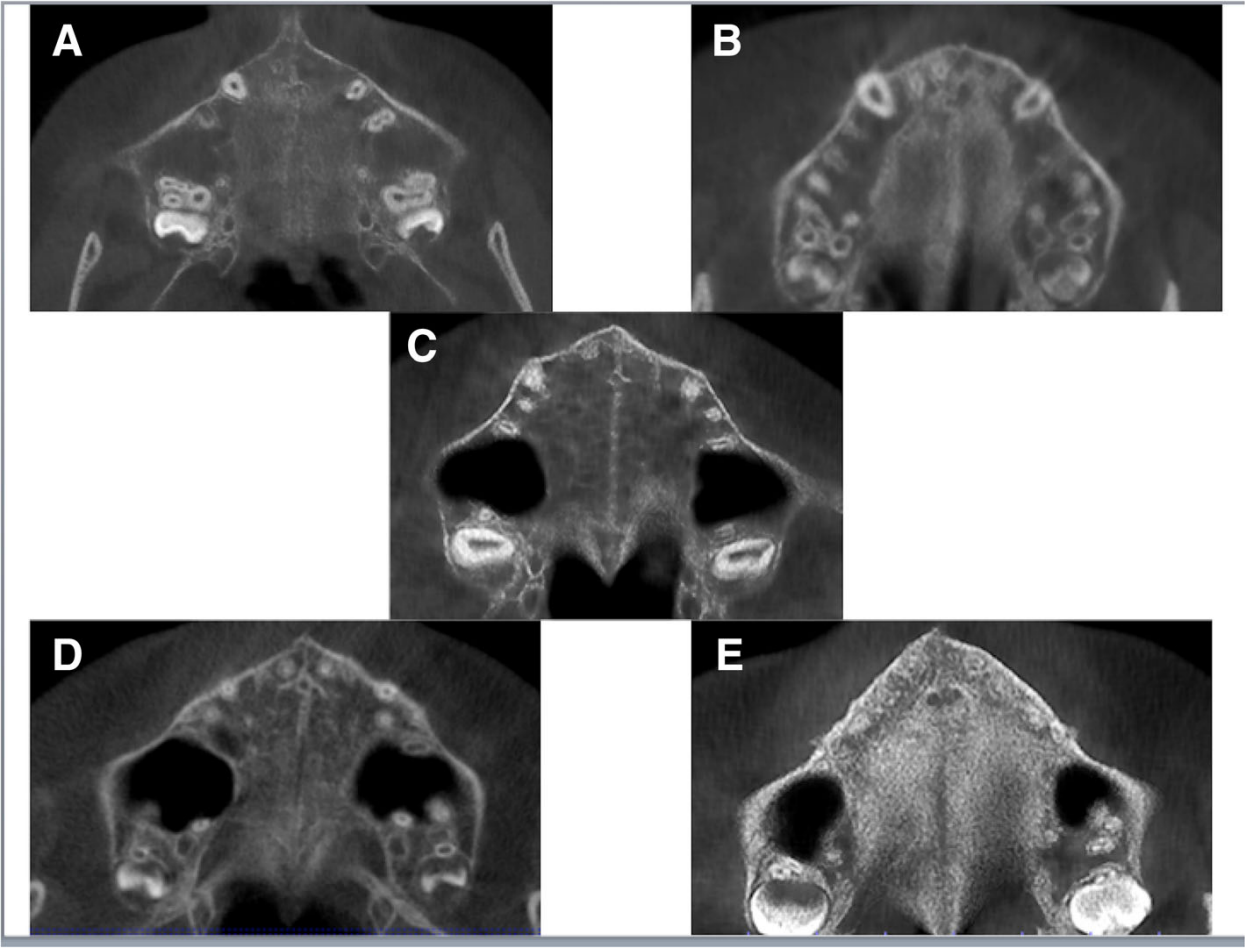
The maturation stages of the midpalatal suture. **a** The midpalatal suture has no interdigitation. **b** The midpalatal suture shows an irregular shape. **c** The midpalatal suture is like as two parallel, scalloped, high-density lines. **d** Fusion of the midpalatal suture has occurred in the palatine bone. **e** Fusion of the midpalatal suture has also occurred in the maxilla

Orientation of the software for assessment was presented in Fig. 1. The maturation stages of the midpalatal suture were as follows and are shown in Fig. 2. The evaluation of CBCT images in our study was performed as same as in the study of Angelieri et al. [19].

*Stage A*: The midpalatal suture is a straight high density line with no interdigitation. *Stage B*: The midpalatal suture shows an irregular shape, like as a scalloped high density line. *Stage C*: The midpalatal suture is like as two parallel, scalloped, high-density lines that are close to each other. *Stage D*: Fusion of the midpalatal suture has occurred in the palatine bone, and in the maxillary portion of the suture, the fusion has not yet occurred. *Stage E*: Fusion of the midpalatal suture has also occurred in the maxilla. The actual suture is not visible, and the bone density is the same as in whole part of the palate.

Sixteen images were randomly selected and midpalatal suture maturation stage reassessed by the same researcher (GS) 2 weeks after the first assessment. According to Houston’s formula, the method error was found as 0.91 [20].

### Statistical analysis

Statistical analyses were performed using the Statistical Package for Social Science (SPSS for Windows, version 23.0, SPSS Inc., Chicago, IL, USA). The normality of the data was assessed by using one-way ANOVA test.

Normally distributed data were evaluated with paired sample *t* tests. The significance level was taken as *P* < 0.05.

## Results

The description of the measurements used in the study is presented in Table 1. Changes of the measurements within the groups are shown in Table 2. Mean significant increases were found between T0 and T1 in all measurements of all groups except interdental width in group E and palatal intermolar width and buccal intermolar width in group B. There were significant differences between all of the measured parameters of pre-peak and post-peak subjects. There were no significant differences between pre-treatment and post-treatment results (Table 3).

**Table 1:** Description of the measurements

Measurements	Description
Interdental width (coronal view)	Interdental width was measured by tracing a line between the mesial points of upper central incisors at the level of cementoenamel junction.
Nasal spine width (coronal view)	Nasal spine width was measured by tracing a line between the right and left most lateral parts of the nasal spine.
Nasal base width (coronal view)	Nasal base width was measured by tracing a line between the right and left most lateral parts of the skeletal nasal base at the level of nasal floor.
Maxillary width (coronal view)	Maxillary width was measured by tracing a line between the left and right jugulare points of the maxilla.
Buccal intermolar width (transversal view)	The distance between the most buccal point of mesiobuccal cusp’s enamel margin of the right and left upper first molars.
Palatal intermolar width (transversal view)	The distance between the most palatal point of mesiopalatal cusp’s enamel margin of the right and left upper first molars.

**Table 2:** Measurements with changes according to midpalatal suture maturation groups

Measurements	Time	Stage A (mean ± SD)	Stage B (mean ± SD)	Stage C (mean ± SD)	Stage D (mean ± SD)	Stage E (mean ± SD)	*P*
Interdental width	T0	1.98 ± 0.67	1.55 ± 0.33	2.27 ± 0.56	1.55 ± 0.82	2.11 ± 0.79	0.234
	T1	2.79 ± 0.38	2.49 ± 0.29	3.02 ± 0.72	2.43 ± 0.57	2.67 ± 0.92	0.411
	*P*	*0.014*	*0.035*	*0.001*	*0.035*	0.091	
Nasal spine width	T0	4.77 ± 1.32	4.13 ± 1.35	4.54 ± 1.34	4.65 ± 1.84	4.25 ± 1.14	0.940
	T1	5.57 ± 1.12	5.14 ± 1.14	5.55 ± 1.47	5.67 ± 1.60	5.29 ± 0.98	0.963
	*P*	*0.011*	*0.045*	*0.002*	*0.018*	*0.032*	
Nasal base width	T0	18.91 ± 3.02	17.95 ± 6.1	16.71 ± 1.9	19.12 ± 5	18.59 ± 4.57	0.774
	T1	20.33 ± 2.78	19.18 ± 5.84	19.2 ± 2.55	21.52 ± 6.03	19.63 ± 4.46	0.845
	*P*	*0.006*	*0.030*	*0.012*	*0.008*	*0.044*	
Maxillary width	T0	60.19 ± 2.15	55.41 ± 3.40	57.65 ± 4.34	60.33 ± 2.84	58.05 ± 3.906	0.127
	T1	63.17 ± 3.05^a^	57.7 ± 1.83^b^	60.78 ± 3.11^ab^	63.68 ± 1.41^a^	60.35 ± 3.8^ab^	*0.012*
	*P*	*0.001*	0.147	*0.001*	*0.015*	*0.009*	
Palatal intermolar width	T0	31.16 ± 3.03	29.77 ± 2.20	30.07 ± 2.89	31.24 ± 1.72	30.03 ± 3.88	0.824
	T1	34.85 ± 3.84	33.34 ± 3.10	34.57 ± 4.33	35.9 ± 2.51	35.08 ± 4.18	0.862
	*P*	*0.005*	0.061	*0.001*	*< 0.001*	*< 0.001*	
Buccal intermolar width	T0	49.75 ± 4.02	48.43 ± 1.97	48.66 ± 2.99	51.27 ± 2.30	48.51 ± 4.73	0.523
	T1	55.07 ± 3.67	51.96 ± 2.55	53.75 ± 4.11	55.29 ± 3.34	54.02 ± 4.37	0.631
	*P*	*< 0.001*	0.089	*< 0.001*	*0.002*	*< 0.001*	

^a, b^There is no difference in the measurements which were marked with the same letter

*SD* standard deviation

**Table 3:** Mean differences between the pre-pubertal and post-pubertal groups

	Time	Pre-pubertal group (*n* = 18)	Post-pubertal group (*n* = 14)	P*
Interdental width	T0	1.98 ± 0.58	1.78 ± 0.82	0.429
	T1	2.82 ± 0.54	2.53 ± 0.71	0.206
	P**	*< 0.001*	*0.005*	
Nasal spine width	T0	4.55 ± 1.28	4.48 ± 1.54	0.895
	T1	5.47 ± 1.22	5.51 ± 1.33	0.933
	P**	*< 0.001*	*0.001*	
Nasal base width	T0	17.84 ± 3.4	18.9 ± 4.61	0.462
	T1	19.64 ± 3.31	20.73 ± 5.29	0.478
	P**	*< 0.001*	*0.001*	
Maxillary width	T0	58.21 ± 3.73	59.38 ± 3.39	0.383
	T1	61.12 ± 3.44	62.3 ± 3.04	0.337
	P**	*< 0.001*	*0.001*	
Palatal intermolar width	T0	30.44 ± 2.76	30.74 ± 2.73	0.769
	T1	34.44 ± 3.77	35.55 ± 3.16	0.396
	P**	*< 0.001*	*< 0.001*	
Buccal intermolar width	T0	49.05 ± 3.2	50.12 ± 3.61	0.389
	T1	53.92 ± 3.69	54.76 ± 3.67	0.536
	P**	*< 0.001*	*< 0.001*	

^*^Unpaired sample *t* test values

^**^Paired sample *t* test values

There were no statistically significant differences in mean differences of each parameter according to the pre- and post-peak groups; these results are given in Table 4.

**Table 4:** Intergroup differences of each parameters

	Pre-peak group	Post-peak group	Test statistics*	*P*
İnterdental width	0.84 ± 0.46	0.75 ± 0.74	0.062	0.675
Nasal spine width	0.93 ± 0.6	1.03 ± 0.76	0.104	0.664
Nasal base width	1.81 ± 1.56	1.83 ± 1.48	0.039	0.960
Maxillary width	2.91 ± 1.68	2.92 ± 2.12	0.001	0.987
Palatal intermolar width	4 ± 2.33	4.82 ± 1.29	0.995	0.211
Buccal intermolar width	4.87 ± 2.22	4.64 ± 1.81	0.143	0.764

*ANOVA test statistics

## Discussion

Orthodontists meet two basic questions while considering rapid maxillary expansion treatment. The first one is the method of expansion (orthopedic or surgical), and the second is the type of expansion device that will be used. The other and perhaps the most important point is skeletal and dental response to the expansion.

Fusion of maxillary sutures is completed at the age of 14–15 in females and 15–16 in males [12]. It is a general belief that rapid maxillary expansion is more skeletal in individuals who are younger these ages. However, the expansion is thought to be dental and dentoalveolar rather than skeletal in the patients older than those ages [20]. Age is usually used as a parameter in the evaluation of maturation of patients; however, skeletal maturation levels are more reliable than chronological age [10, 19].

Pubertal stage of a patient could be determined by using hand-wrist radiography or cervical vertebral maturation method, but when it comes to predict the response to maxillary expansion, the midpalatal suture maturation level was thought to be more related with this treatment. It was previously evaluated in histological and radiological studies, and in histological studies, it was found that various maturation levels were present in the same age group [21–23]. Also, Angelieri et al. [19] stated that the children in same chronological age could present differences in midpalatal suture maturation levels.

Bacetti et al. [24] used the CVM method and classified the patients by using the said method [23] as pre-peak and post-peak subjects to compare the outcomes of RME treatment between these two growth phases. Furthermore, Angelieri et al. [19] have noticed the need of an assessment method for the individual midpalatal suture maturation stage and determined a classification method. Angelieri et al. [25] noted that pre-pubertal CVM stages of CS1, CS2, and CS3 are equal to midpalatal maturational stages of A, B, and C, respectively. This statement was found as reliable for CS5 and stages D and E. Therefore, the aim of this study was to evaluate the dento-skeletal changes after RME in patients who are classified as pre-peak and post-peak based on midpalatal suture maturation method and to determine if there was a difference between the two groups.

When maxillary expansion is performed, a resistance originates from the neighborhood structures of maxilla such as apertura piriformis, zygomatic buttress, and ptrygoid plate, zygomaticotemporal, zygomaticomaxillary, pterygopalatine sutures, and also, the main target is the midpalatal suture itself [26]. The differences in individual maturation levels of the midpalatal suture produce different treatment results. A few methods were previously used to evaluate the midpalatal suture maturation such as midpalatal suture density (MPSD), obliteration index (OI), and midpalatal suture morphology (MPSM), and these methods were found more reliable than chronological age [9, 10, 19, 25]. But there were some limitations of these methods. The obliteration index was introduced to make a decision between the treatment alternatives of surgical assisted maxillary expansion and orthopedic one, but it was reported that there could be even no obliteration of midpalatal suture in older adult patients [24]. Measurement of the midpalatal suture density was found as a reliable method for estimating the skeletal outcomes of RME, but this method is based on the settings of CBCT machine, and standardization of the different types of the machine is difficult [27].

To overcome standardization errors and determine bone density, Cassetta et al. [28] suggested using a conversion method for transforming the gray density values (voxel values) of CBCT to Hounsfield unit (HU) values of computed tomography.

Angelieri et al. [19] stated that dividing the maturation stages as A–C and D and E could be useful to make a choice between orthopedic expansion and surgical one. In groups A–C, more skeletal expansion could be managed by using RME appliance, but in the more maturated groups, D and E, more dental bending might occur and surgical assisted expansion option can be an alternative. To determine the differences in skeletal and dental findings between the pre-peak and post-peak individuals, we also divided the groups as adviced by Angelieri et al [19].

Opening of a median diastema between the upper central incisors is an expected outcome of RME treatment. It is admitted that a direct relationship is present between the degree of the diastema and the amount of orthopedic expansion, clinically [29].

In this study, significant increase in interdental width after RME was found in all of the midpalatal maturational stages except the stage E group. In stage E, the maturation of midpalatal suture is completed and this might be the reason of the lower amount of change.

It is presented in some previous studies that size of nasal structures is affected by the expansion of maxilla [30, 31]. This was in consistency with our study; furthermore, nasal spine width and skeletal nasal base width showed significant increases in all of the sutural maturation groups and these increases were present in both of the pre-puberty and post-puberty groups. Similar to our findings, Bacetti et al. [24] noted that the increase in nasal cavity width was significantly higher in the pre-peak group in short time after RME than that of the post-peak patients.

In their study, Pereira et al. [32] found a maxillary width increase with a mean value of 1.76 mm while Capalette et al. [33] presented this value as 3.6 mm. In our study, this value was 2.91 mm for the pre-peak and 2.92 mm for the post-peak group, and the increase in maxillary width was significant in both of the two groups.

In our study, maxillary width, palatal intermolar width, and buccal intermolar width were increased after RME treatment in all of the suture maturation groups except group B. However, this difference was not significant in comparison with the other midpalatal suture maturation groups. Furthermore, the change of these parameters significantly increased in the pre-peak and post peak-groups but the difference was not significant for the two groups. We think that use of the same RME appliance with the same procedure might be resulted similar orthopedic and orthodontic expansion.

The mean increase in skeletal width of the maxilla was found as lower than that of the width of molar teeth in buccal and palatinal measurements, but this result might have been related to the inclination of molar and pre-molar teeth. As reported previously, the inclination of posterior teeth is admitted as a normal situation for RME treatment and this was the same with our study [6, 32, 34–36].

Bacetti et al. [17] concluded that application of RME treatment in the pre-peak phase of puberty resulted more accentuated transversal skeletal changes than that of the post-peak phase. They also stated that application of RME in the post-peak phase resulted in more dentoalveolar changes; however, we could not find significant differences between the groups in our study. Although RME application resulted as an increase in all of the parameters, there were no differences found between the pre-puberty and post-puberty groups.

## Conclusions

There were no differences found between the dental and skeletal changes between the pre-peak and post-peak groups.

Maxillary width was significantly less in group B than that of the other midpalatal suture maturation groups.

It could not be demonstrated that it is possible to predict the amount of maxillary expansion according to the midpalatal suture maturation level.

The results of this study can be assessed as valid or true until 18 years old.

If the distribution of the males and females was equal in each midpalatal suture maturation group, the effect of gender differences on results would be minimized.

## Data Availability

The raw data is present in the CBCT software of our university clinic.

## Abbreviations

CBCT: Cone beam computed tomography
HU: Hounsfield unit
post-peak: Post-pubertal peak
pre-peak: Pre-pubertal peak
RME: Rapid maxillary expansion
T0: Pre-treatment stage
T1: Post-treatment stage
